# Integrated Bioinformatics Algorithms and Experimental Validation to Explore Robust Biomarkers and Landscape of Immune Cell Infiltration in Dilated Cardiomyopathy

**DOI:** 10.3389/fcvm.2022.809470

**Published:** 2022-04-01

**Authors:** Qingquan Zhang, Mengkang Fan, Xueyan Cao, Haihua Geng, Yamin Su, Chunyu Wu, Haiyan Pan, Min Pan

**Affiliations:** ^1^Department of Cardiology, Affiliated Hospital of Nantong University, Nantong, China; ^2^Department of Obstetrics and Gynecology, Affiliated Hospital of Nantong University, Nantong, China; ^3^Department of Cardiology, West China (Sanya) Hospital, Sichuan University, Sanya, China

**Keywords:** dilated cardiomyopathy, *CD163*, biomarkers, immune, bioinformatic algorithms

## Abstract

**Background:**

The etiology of dilated cardiomyopathy (DCM) is unclear. Bioinformatics algorithms may help to explore the underlying mechanisms. Therefore, we aimed to screen diagnostic biomarkers and identify the landscape of immune infiltration in DCM.

**Methods:**

First, the CIBERSORT algorithm was used to excavate the proportion of immune-infiltration cells in DCM and normal myocardial tissues. Meanwhile, the Pearson analysis and principal component analysis (PCA) were used to identify immune heterogeneity in different tissues. The Wilcoxon test, LASSO regression, and machine learning method were conducted to identify the hub immune cells. In addition, the differentially expressed genes (DEGs) were screened by the limma package, and DEGs were analyzed for functional enrichment. In the protein–protein interaction (PPI) network, multiple algorithms were used to calculate the score of each DEG for screening the hub genes. Subsequently, external datasets were used to further validate the expression of hub genes, and the receiver operating characteristic (ROC) curve was used to analyze the diagnostic efficacy. Finally, we examined the expression of hub biomarkers in animal models.

**Results:**

A total of 108 DEGs were screened, and these genes may be related to biological processes such as cytolysis, positive regulation of cytokine secretion, etc. Two types of hub immune cells [activated natural killer (NK) cells and eosinophils] and four hub genes (*ASPN, CD163, IL10*, and *LUM*) were identified in DCM myocardial tissues. *CD163* was verified to have the capability to diagnose DCM with the most excellent specificity and sensitivity. It is worth mentioning that the combined *CD163* and eosinophils may have better diagnostic efficacy. Moreover, the correlation analysis showed *CD163* was negatively correlated with activated NK cells. Finally, the results of the mice model also indicated that *CD163* might be involved in the occurrence of DCM.

**Conclusion:**

*ASPN, CD163, IL10*, and *LUM* may have a potential predictive ability for DCM, and especially *CD163* showed the most robust efficacy. Furthermore, activated NK cells and eosinophils may relate to the occurrence of DCM.

## Introduction

Dilated cardiomyopathy (DCM) is a type of primary heart disease characterized by dilation of the ventricular cavity and impaired contraction of the myocardium (1). DCM is structural heart disease, and despite some advances in treatment and diagnosis, the prognosis for such patients is still poor (2). Especially in clinical practice, there is a lack of robust biomarkers to predict the occurrence and progression of DCM. Meanwhile, the change of immune cells in the pathological process of DCM tissues has not been explored. It suggests that we should explore the underlying occurrence and development mechanism of DCM, which will help us with clinical decision-making.

With the development of high-throughput sequencing technology, bioinformatics analysis plays an important role in medical research, which provides an objective basis for medical scientists to explore the pathogenesis of various diseases (3). This study is based on the structural cardiomyopathy sequencing datasets from the Myocardial Applied Genomics Network (MAGNet) (4), which has the largest sample size of DCM patients to date. We aimed to screen the hub gene and immune cell infiltration in the DCM, which provided new ideas and potential therapeutic targets for the treatment in the future.

## Materials and Methods

### Dilated Cardiomyopathy Datasets

GSE141910 from MAGNet, obtained by download from the Gene Expression Omnibus (GEO) database (5), contains transcriptomic data and corresponding clinical information. The dataset was annotated using the GPL16791 platform files. The dataset included 116 DCM samples, 28 hypertrophic cardiomyopathy (HCM) samples, and 166 normal samples, all of which were derived from left ventricle (LV) myocardial tissue. In this study, we included only normal samples and DCM samples for bioinformatics analysis. In addition, GSE42955 and GSE79962 were defined as external datasets. GSE79962 was annotated using the GPL6244 platform, including 11 normal samples and 9 DCM samples; GSE42955 was also annotated by the GPL6244 platform, including 5 normal samples and 12 DCM samples.

### Screening of Hub Immune Cells

The CIBERSORT algorithm (6) was used to calculate the proportion of different immune cell types based on the expression level of immune cell-related genes. The output results of 22 infiltrating immune cells were integrated to generate a matrix of immune cell fractions for analysis. First, we used the Wilcoxon test for screening different immune cells between DCM and normal samples. Meanwhile, 10-fold cross-validation of LASSO-logistic was used to further screen the characteristic cells [“glmnet” package in R software (7)]. Finally, support vector machine-recursive feature elimination (SVM-REF), which is a machine learning method based on support vector machines, finds the best cells by deleting feature vectors generated by SVM (8) (“e1071” and “msvmRFE” packages in R software). The intersection of immune cells from the above three methods was identified as final hub immune cells.

### Screening of Differentially Expressed Genes and Enrichment Analysis

The “limma” package (9) in R software was used to screen differentially expressed genes (DEGs) between DCM and normal samples, with a threshold set to |logFC| >2 and adj. *p* < 0.05. Moreover, enrichment analysis was performed in genes from the hub module using “clusterProfiler” package in R software (10). The results of Gene Ontology (GO) analysis (11) and the enrichment analysis of Kyoto Encyclopedia of Genes and Genomes (KEGG) (12) were extracted from the R software.

### Screening of Hub Genes and Exploring Predictive Value

The protein–protein interaction (PPI) network composition of the DEGs was determined by a threshold score >0.4. Genes in the PPI network were then analyzed using 12 algorithms built into the cytoHubba (13) plug-in in Cytoscape software (14). The genes scored by each algorithm were used to screen hub genes by the “UpSet” package in R software. In addition, the area under the ROC curve (AUC) value is considered as an indicator to evaluate the effectiveness of biomarkers.

### Animal Experiment

A total of 20 C57BL/6 male mice (6 weeks old) were purchased from the Model Animal Research Center of Nantong University and were fed a normal diet under specific pathogen-free (SPF) conditions. All animal procedures were in accordance with the Care and Use of Laboratory Animals published by the National Institutes of Health and approved by the Institutional Ethics Committee of Affiliated Hospital of Nantong University. In addition, all mice were randomly divided into two groups, namely, the control group and the doxorubicin (DOX) group. The DOX groups were established as a DCM model by administration of doxorubicin hydrochloride (Absin, China) *via* intraperitoneal injection (4 mg/kg) every other week for a cumulative dose of 12 mg/kg.

### Echocardiography

Cardiac function was assessed by transthoracic echocardiography (VisualSonics, USA) 2 weeks later. Mice were anesthetized with isoflurane (1.5% in air) and monitored for respiratory frequency and temperature. Echocardiography dimensions (wall thickness and chamber diameter) were obtained using software included in the VisualSonics system.

### Immunofluorescence and Histopathology Analysis

For immunofluorescence analysis, after incubation with secondary antibody (Servicebio, GB11340-1) and counterstaining with 4′,6-diamidino-2-phenylindole (DAPI), images were observed and attained by a fluorescent microscope (15). More incubation details were described in previous studies. Cardiac tissues from different groups were preserved for 24 h in 4% paraformaldehyde. Tissues that had been paraffin-embedded were cut into 6 μm pieces and dried at 45°C. For hematoxylin and eosin (HE) staining, the tissues were stained with hematoxylin (3 min) and then placed in 1% hydrochloric acid in ethanol (2 s). After 3 min of eosin staining, the slices were dehydrated with gradient alcohol, permeabilized with xylene, sealed with resinene, and dried for 72 h. For Masson staining, the tissue pieces were dewaxed three times with xylene for 5 min each time before being immersed in an alcohol gradient. The Masson Kit (Solarbio, G1340) was used to stain the parts. Light microscopy was used to examine changes in inflammatory infiltration and myocardial fibrosis. For TUNEL assay, we used TUNEL Assay Kit (Servicebio) evaluated cardiomyocyte death. Nuclei of normal and apoptotic cells appeared light blue and yellow-brown after staining, respectively. The number of apoptotic cells was divided by the total number of cells in each field of view (×400) to get the apoptotic index value. Images were analyzed using Image-Pro Plus software.

### Statistical Analysis

All statistical analyses were performed using R software (v.4.0.1). Detailed statistical methods about transcriptome data processing are covered in the above section. *p* < 0.05 was considered statistically significant.

## Results

### Landscape of Immune Cell Infiltration in DCM and Normal Myocardium Tissues

**Figure 1:**
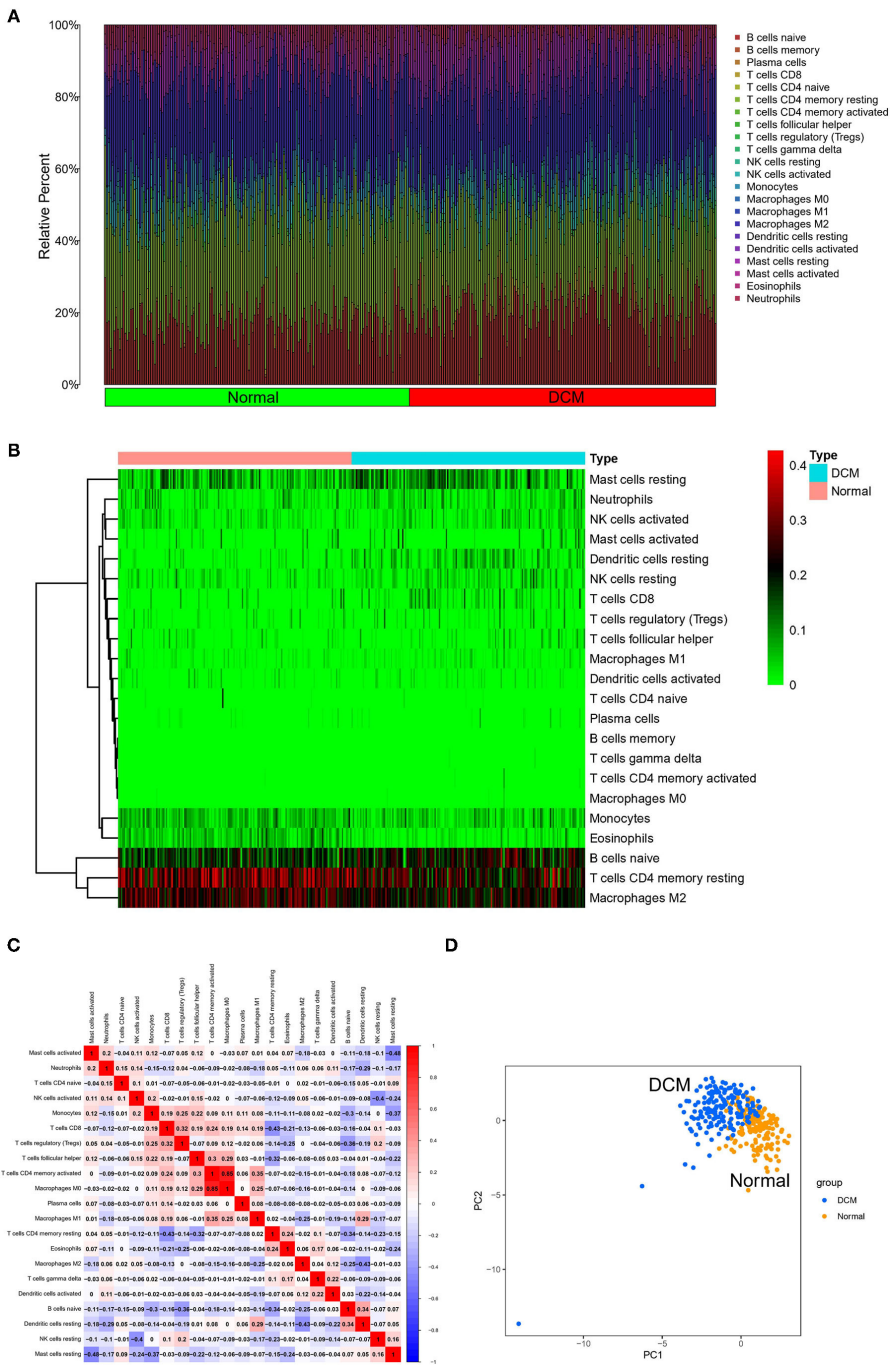
Landscape of immune cell infiltration. The histogram **(A)** and heatmap **(B)** about expression of 22 types of immune cells in DCM and normal samples. Different colors represent different immune cells in the histogram. **(C)** Heatmap of correlation analysis of entire immune cells in DCM tissue samples. Blue represents negative correlation and red represents positive correlation. **(D)** PCA based on immune cells. Blue represents DCM samples and orange represents normal samples. DCM, dilated cardiomyopathy; PCA, principal component analysis.

First, immune cell infiltration was explored in 166 normal myocardium tissues and 116 DCM tissues. Most of the samples met the threshold of the CIBERSORT analysis (*p* < 0.05). Different colors in the histogram represented the proportion of different immune cells (Figure 1A), and the color depth of the heatmap represented the expression level of immune cells (Figure 1B). The results showed that resting mast cells, neutrophils, activated natural killer (NK) cells, resting dendritic cells, resting NK cells, CD8 T cells, monocytes, eosinophils, B-cell naive, resting memory CD4 T cells, and M2 macrophages might be the primary immune infiltrating cells in myocardium tissues. In the correlation analysis, in particular, M0 macrophages and activated CD4 memory T cells showed the strongest positive correlation (*r* = 0.85). However, resting mast cells and activated mast cells showed the strongest negative correlation (*r* = −0.48) in DCM myocardium tissues (Figure 1C). Interestingly, we were able to completely distinguish DCM and normal myocardium tissues by PCA based on the analysis results of CIBERSORT, suggesting the critical role of immune mechanisms in the pathogenesis of DCM (Figure 1D).

### Identification of Hub Immune Cells

**Figure 2:**
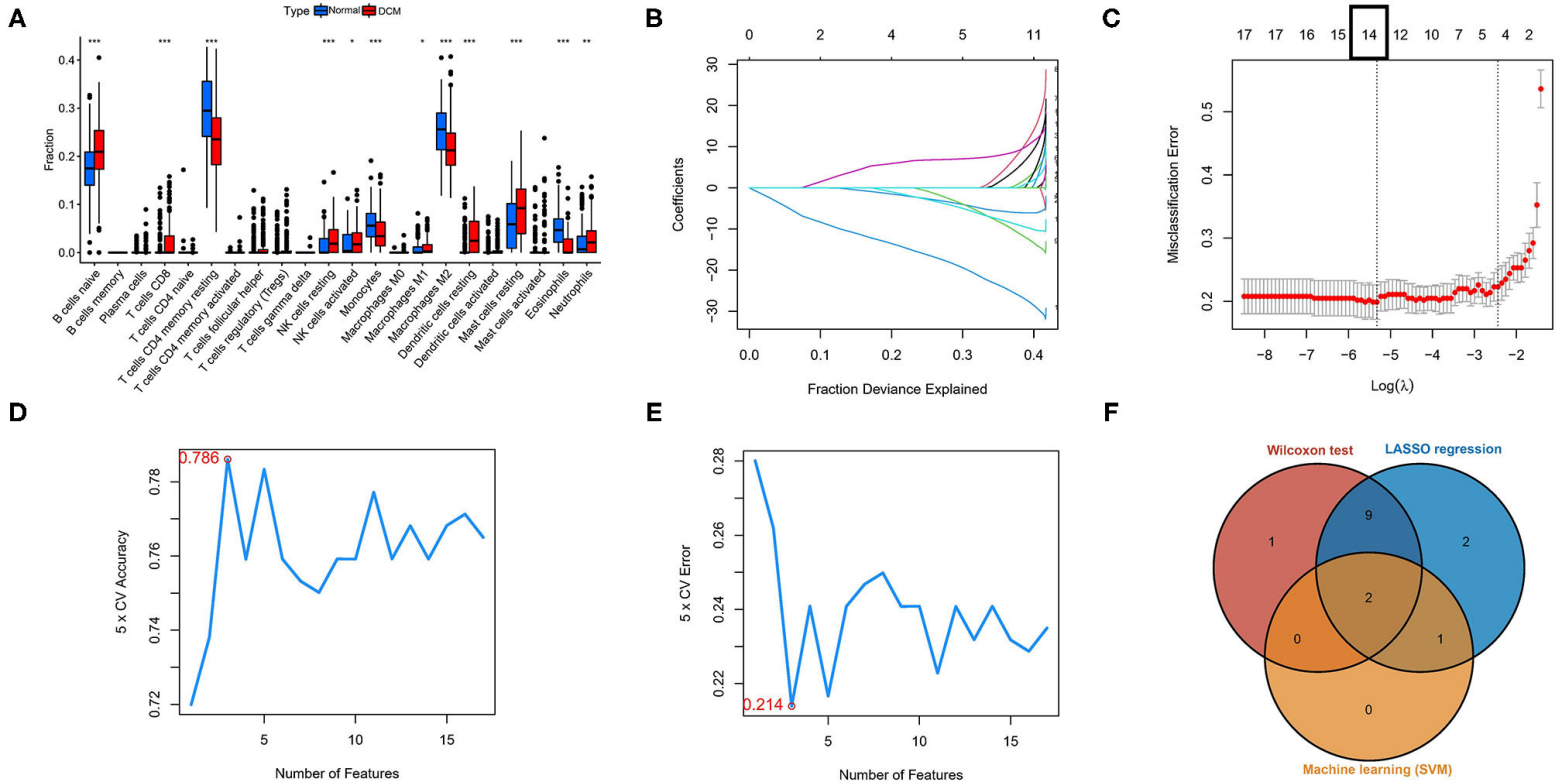
Identification of hub immune cells by three bioinformatics algorithms. **(A)** Different immune cells in different tissues by the Wilcoxon test. **(B,C)** The LASSO-logistic regression analysis for 17 immune cells. Accuracy value **(D)** and error value **(E)** in SVM-REF method. **(F)** A Venn plot for intersecting the results of three methods. *p < 0.05, **p < 0.01, ***p < 0.001. SVM-REF, support vector machine-recursive feature elimination.

Three different algorithms, the Wilcox test, LASSO-logistic regression, and SVM-REF, were used to identify the hub immune cells in different tissues. In the difference analysis, a boxplot showed that 13 types of immune cells in different tissues were significantly different (*p* < 0.05), as shown in Figure 2A. Meanwhile, to ensure the accuracy of LASSO-logistic and SVM-REF, we removed some immune cells whose expression levels were zero in the vast majority of samples and finally included 17 immune cells for the subsequent two algorithms. The results of the LASSO-logistic are presented in Figures 2B,C, which contained 14 types of immune cells. In the results of SVM-REF, only three types of immune cells were screened with accuracy value = 0.786 and error value = 0.214 (Figures 2D,E). Finally, the two intersecting immune cells (activated NK cells and eosinophils) of the three methods were extracted (Figure 2F).

### Screening of DEGs and Discovery of Underlying Biological Functions

**Figure 3:**
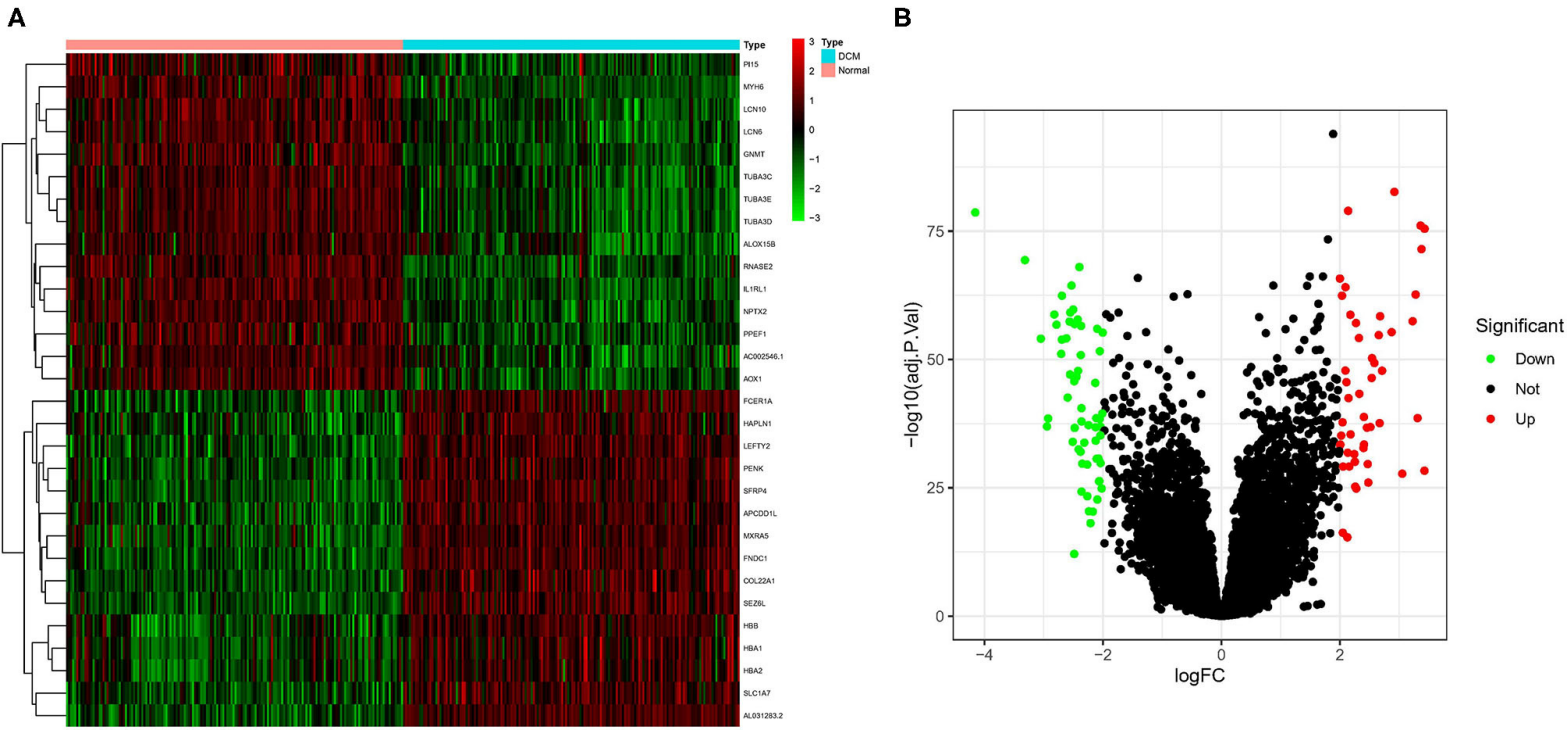
Screening of differentially expressed genes (DEGs) by the limma package. **(A)** The heatmap of DEGs in different tissues; **(B)** the volcano plot of DEGs in different tissues. Different colors indicate whether DEGs is upregulated or downregulated compared to normal myocardium.

**Figure 4:**
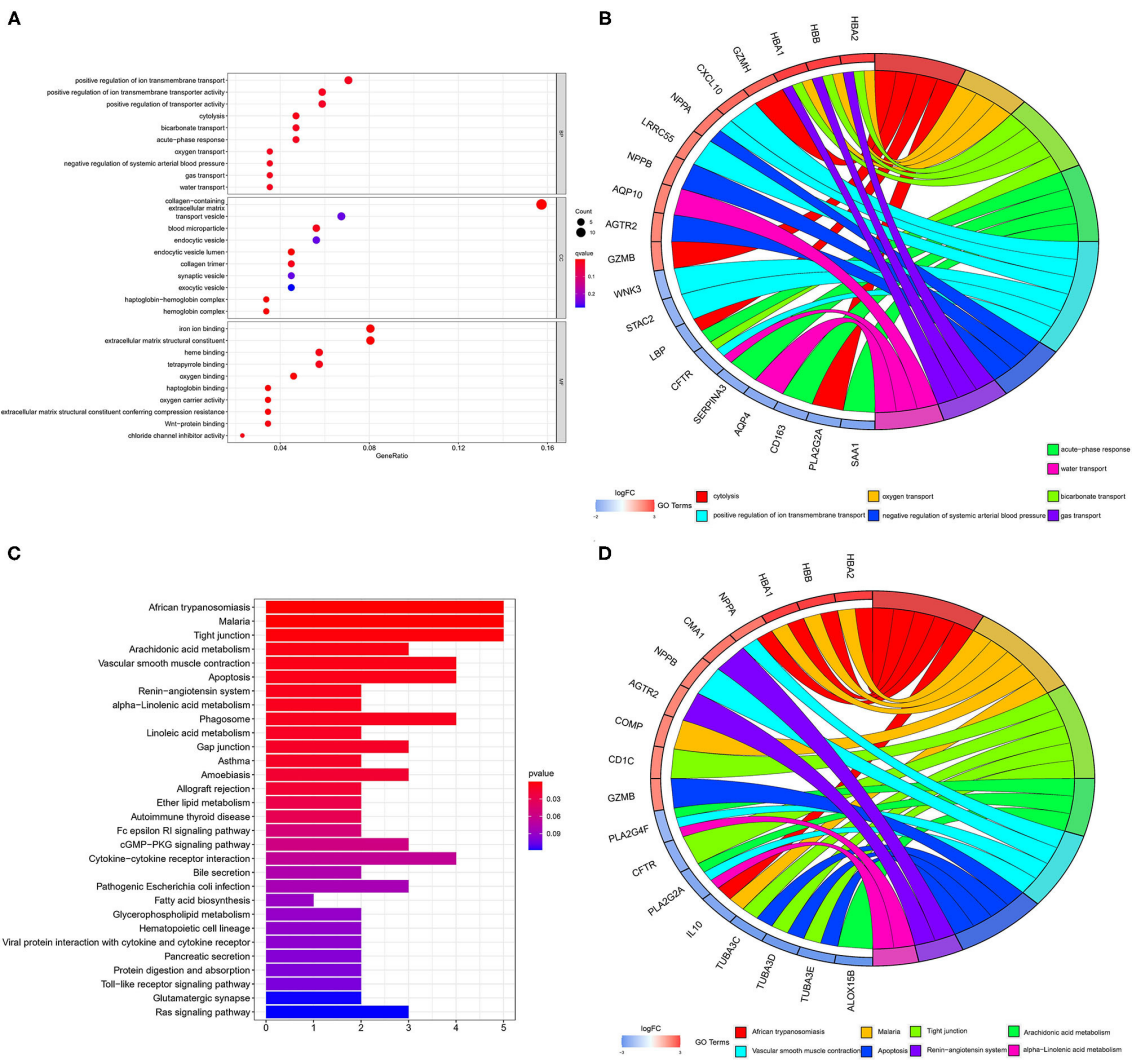
GO and KEGG analyses of DEGs. The results of GO analysis are presented by bar plot **(A)** and circle charts **(B)**. The results of KEGG analysis are presented by bar plot **(C)** and circle charts **(D)**. DEGs, differentially expressed genes; GO, Gene Ontology; KEGG, Kyoto Encyclopedia of Genes and Genomes.

The color depth of the heatmap represented the expression level of DEGs (Figure 3A, Supplementary File 1), and different colors in the volcano plot indicated whether DEGs are upregulated (49 genes) or downregulated (59 genes) compared to normal myocardium (Figure 3B). Subsequently, we performed GO and KEGG enrichment analyses of the above genes. The GO analysis was divided into three parts: cellular component (CC), biological process (BP), and molecular function (MF); in our results, the DEGs were mainly associated with cytolysis, positive regulation of ion transmembrane transport, oxygen transport, etc. (Figures 4A,B). Meanwhile, the DEGs were mainly associated with immune-related and metabolism-related signaling pathways, such as cytokine–cytokine receptor interaction, arachidonic acid metabolism, alpha-Linolenic acid metabolism, etc (Figures 4C,D).

### Identification and Validation of Hub Genes

**Figure 5:**
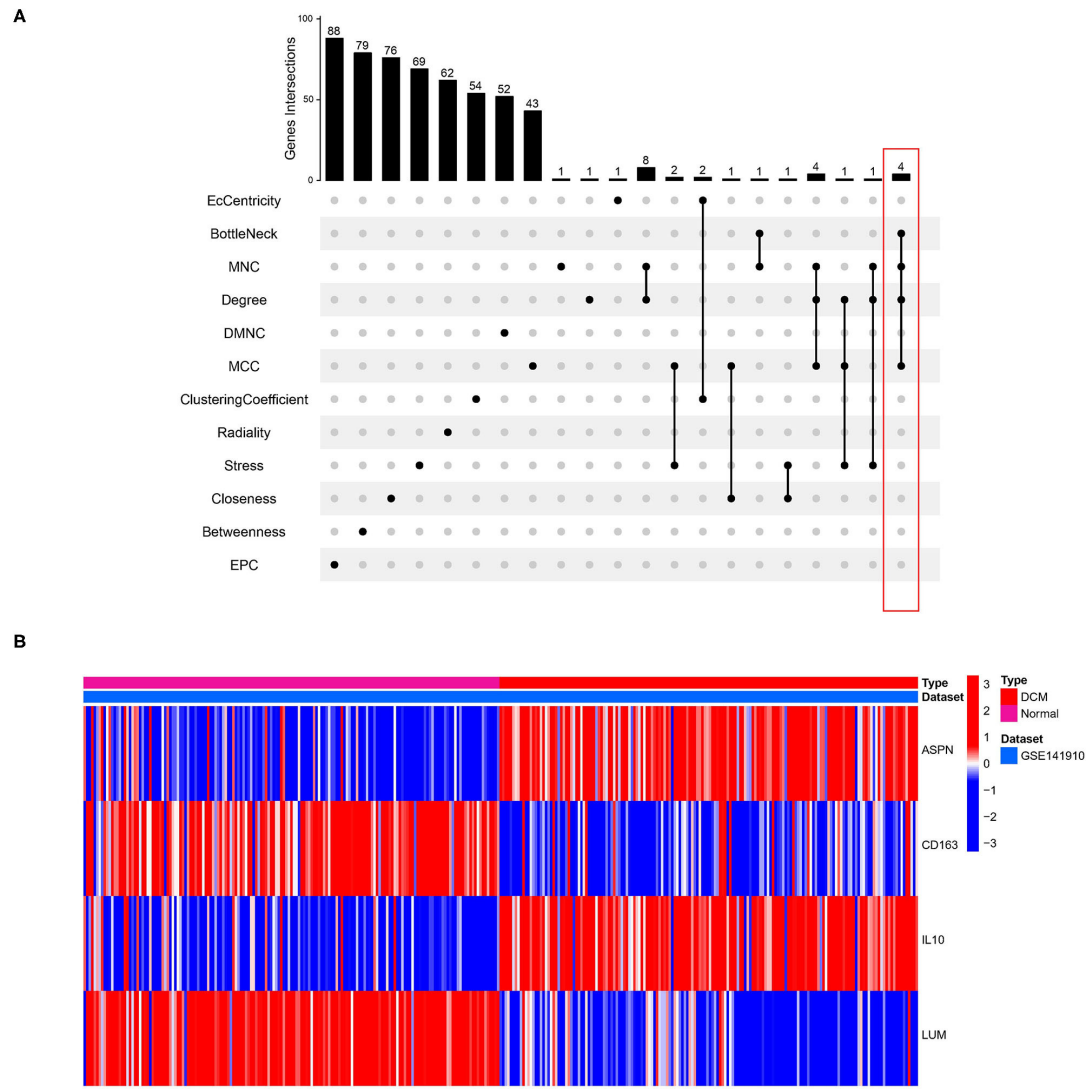
Identification of hub genes. **(A)** Twelve algorithms to screen hub genes. **(B)** Expression of four hub genes n screening dataset are shown by heatmap. Different colors indicate whether genes is upregulated or downregulated compared to normal myocardium.

**Figure 6:**
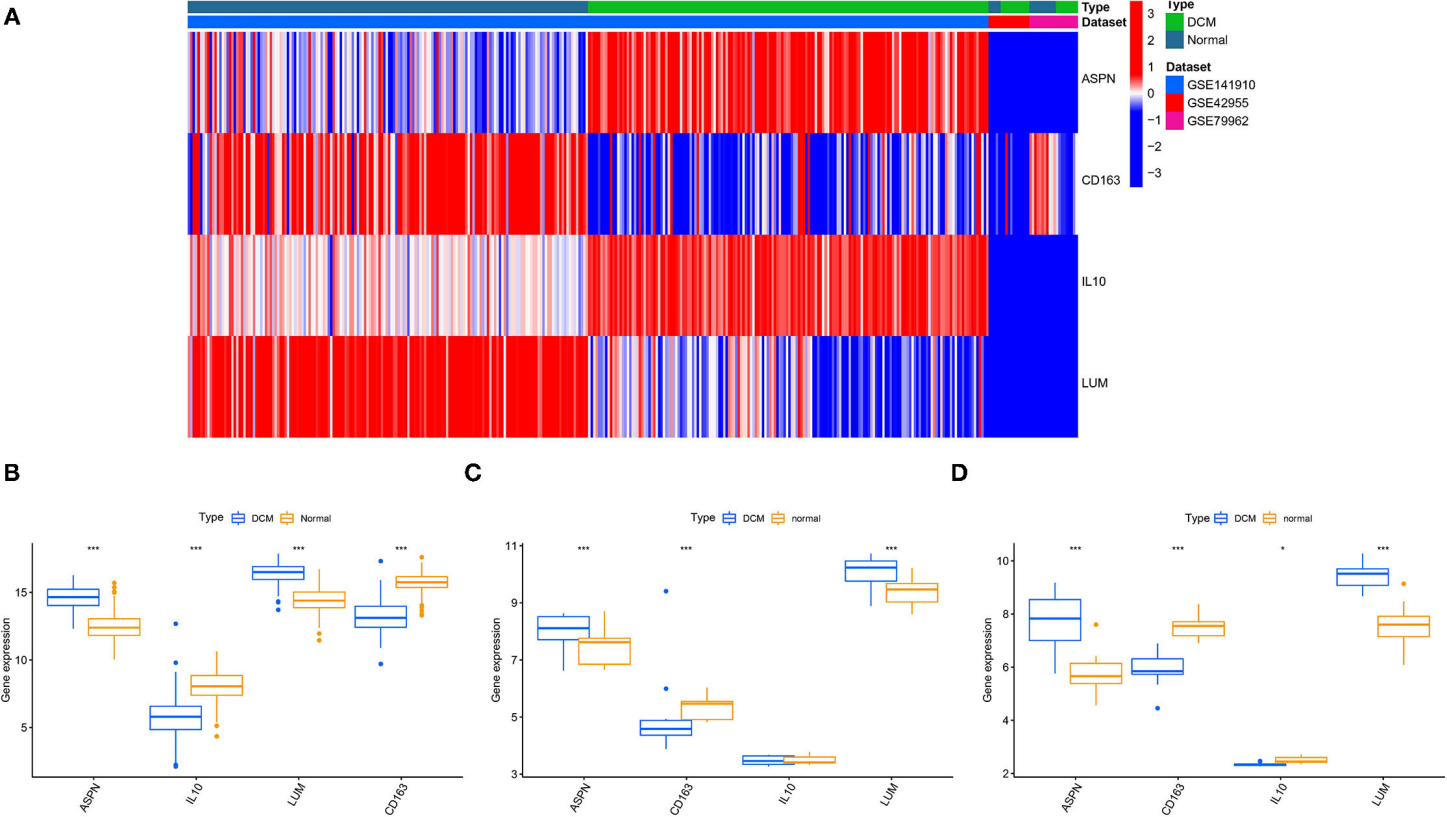
Validation of hub genes expression. **(A)** A composite expression heatmap combining validation and screening datasets. Boxplots for exploring hub genes difference between dilated cardiomyopathy (DCM) and normal samples, including screening dataset **(B)**, GSE42955 dataset **(C)**, and GSE79962 dataset **(D)**. *p < 0.05, **p < 0.01, ***p < 0.001.

We used 12 algorithms to calculate the score of each gene for DEGs in the PPI network. Finally, we screened four hub genes according to the number of intersections (Figure 5A). The expression levels of four hub genes were shown using heatmap, with red and blue representing upregulation and downregulation, respectively (Figure 5B). In order to verify the reliability of the results, GSE42955 and GSE79962 were used for verification. The expression levels of *ASPN, CD163, IL10*, and *LUM* are shown in a composite heatmap (Figure 6A). In the screening dataset, *ASNP* and *LUM* were upregulated in DCM compared to normal samples, whereas *IL10* and *CD163* are downregulated in DCM (Figure 6B). The same trend was also observed in the validation datasets (Figures 6C,D). However, there was no difference in the expression of only *IL10* in the GSE42955 dataset.

### ROC Analysis of Biomarkers

**Figure 7:**
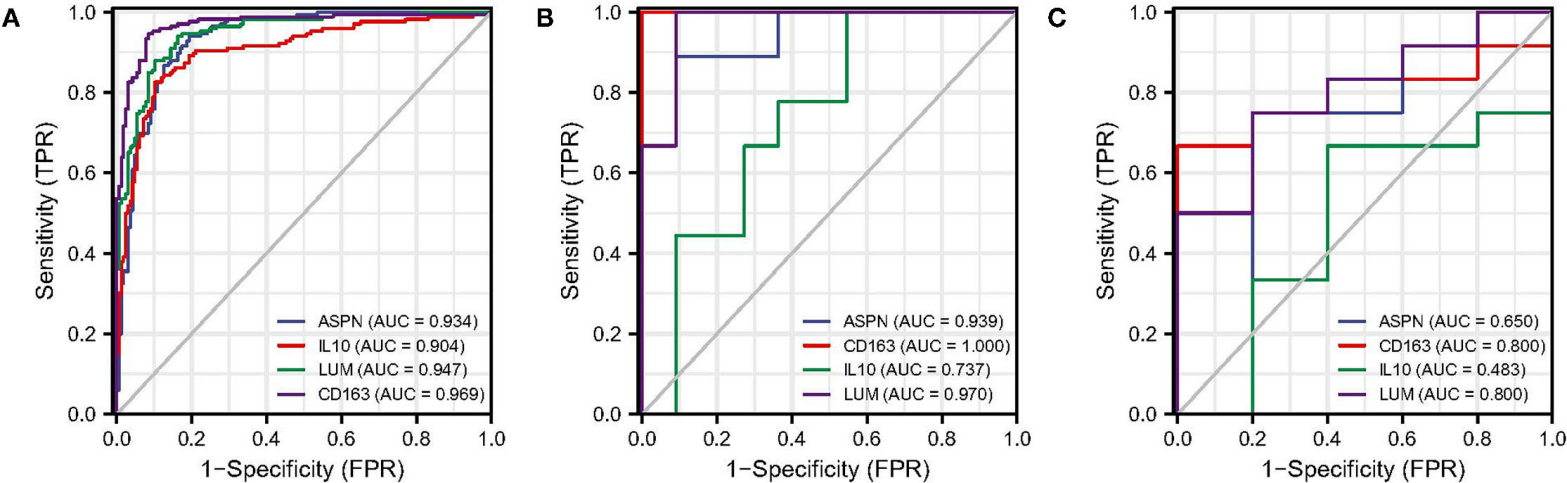
ROC analysis of biomarkers. **(A)** ROC analysis of each hub genes in screening dataset. **(B)** ROC analysis of each hub genes in GSE42955 dataset. **(C)** ROC analysis of each hub genes in GSE79962 dataset. ROC, receiver operating characteristic.

The ROC analysis was used to evaluate the diagnostic efficacy of the four biomarkers for DCM in the screening and validation datasets. In screening dataset, the AUC values of *ASPN, IL10, LUM*, and *CD163* were 0.934, 0.904, 0.947, and 0.969, respectively (Figure 7A). In GSE42955 dataset, the AUC values of *ASPN, IL10, LUM*, and *CD163* were 0.939, 1.000, 0.737, and 0.970, respectively (Figure 7B). In GSE79962 dataset, the AUC values of *ASPN, IL10, LUM*, and *CD163* were 0.650, 0.800, 0.483, and 0.800, respectively (Figure 7C). Thus, *CD163* is the most robust diagnostic marker in the three datasets. We also evaluated the diagnostic performance of two hub immune cells in the screening dataset. The result showed that eosinophils have the best prediction efficiency (AUC = 0.800), whereas activated NK cells (AUC = 0.561) were slightly weaker (Supplementary Figure 1A). Moreover, the combined AUC of eosinophils and *CD163* reached 0.973, as shown in Supplementary Figure 1B.

### Correlation Between Biomarkers and Hub Immune Cells in DCM

**Figure 8:**
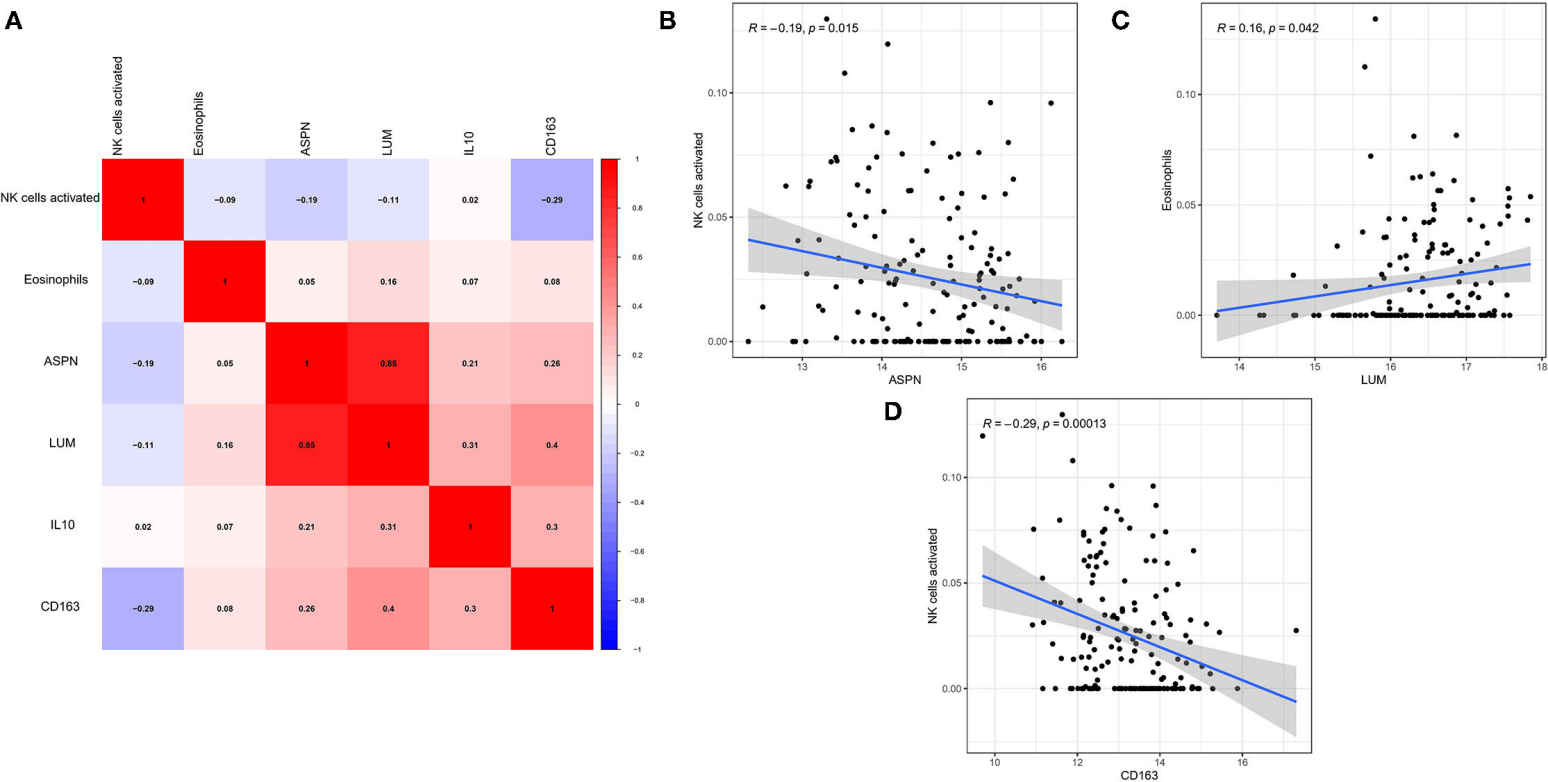
Correlation between biomarkers and hub immune cells. **(A)** The correlation heatmap among four hub genes and two hub immune cells. **(B–D)** Significantly related biomarkers and immune cells with *p* < 0.05.

The correlation among four hub genes and two hub immune cells were analyzed in DCM tissues, as shown a heatmap (Figure 8A). In particular, *ASPN* and *LUM* showed the strongest positive correlation (*r* = 0.85), whereas *CD163* and activated NK cells showed the strongest negative correlation (*r* = −0.29). Meanwhile, we showed the correlation data with *p* < 0.05 more specifically. *ASPN* was negatively correlated with activated NK cells (*r* = −0.19, Figure 8B), *LUM* was positively correlated with eosinophils (*r* = 0.16, Figure 8C), and *CD163* was negatively correlated with activated NK cells (*r* = −0.29, Figure 8D).

### Validation of the *CD163* in DCM Model

**Figure 9:**
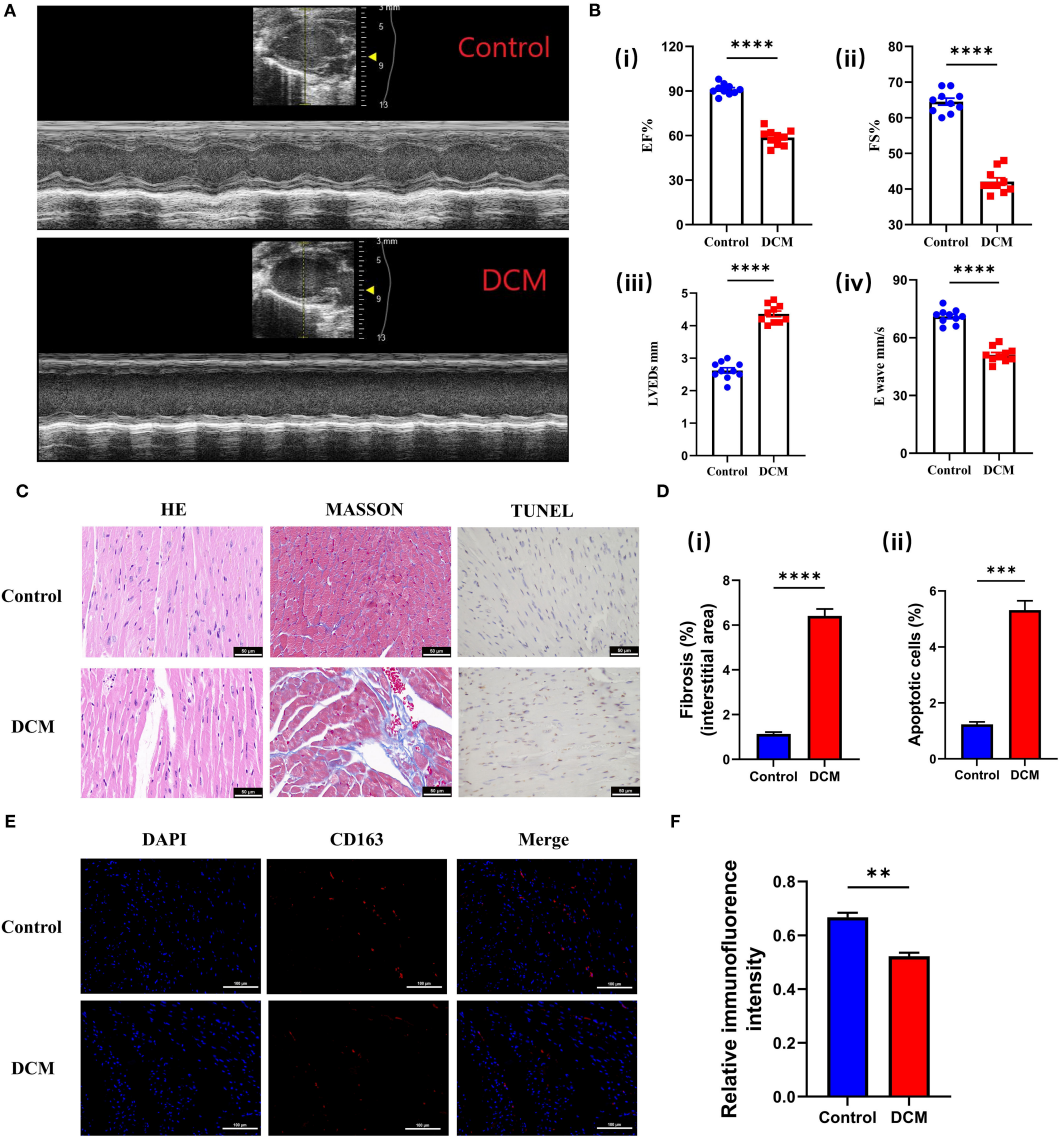
Validation of the *CD163* expression in animal models. **(A)** Representative echocardiography images of mice treated with saline and DOX. The up panel is the control group, and the down panel is the DCM group. **(B)** (i) The ejection fraction, (ii) fractional shortening, (iii) left ventricular end-diastolic diameter, and (iv) E-waves were calculated according to the echocardiography. **(C)** Representative micrographs of HE, Masson, and TUNEL staining (scale: 50 μm). The up panel is the control group, and the down panel is the DCM group. **(D)** (i) The fibrosis ratio (%) in different groups. (ii) The apoptotic cells ratio (%) in different groups. **(E)** Representative fluorescent staining images of *CD163* from cardiac tissues of different models (scale: 100 μm). The up panel is the control group, and the down panel is the DCM group (DAPI, *CD163*, and merge). **(F)** Relative immunofluorence intensity of *CD163* in different groups. The data are presented as the mean ± standard deviation (SD). *p < 0.05, **p < 0.01, ***p < 0.001, ****p < 0.0001. DOX, doxorubicin; DCM, dilated cardiomyopathy; DAPI, 4′,6-diamidino-2-phenylindole; HE, hematoxylin and eosin.

The echocardiographic evaluation revealed that DOX induced a significant reduction of cardiac contractility (Figure 9A), manifested by reduced ejection fraction (EF), ES, left ventricular end-systolic dimension (LVEDs), and E wave, compared with the control group (Figure 9B). Meanwhile, different staining results also suggested that fibrosis and apoptosis cells were significantly increased in DCM mice (Figures 9C,D). The above experimental results showed that our animal model was successful. Therefore, we used immunofluorescence to detect *CD163* expression in different mice. Excitingly, the alterations in the expression of *CD163* detected in mice matched bioinformatics results (Figures 9E,F), suggesting a possible connection between *CD163* and DCM.

## Discussion

Dilated cardiomyopathy is one of the leading causes of sudden death and heart failure. However, the etiology of DCM is not fully understood (1). However, some genetic markers with high mutation frequency have been identified by second-generation sequencing technology. *TTN* (16) and *LMNA* (17) genes are generally considered to play an important role in the development of DCM. But the abnormal expression of other genes and their association with different types of immune cells have not been further discussed.

We identified *ASPN, CD163, IL10*, and *LUM* as the hub genes in the DCM development, and *CD163* was verified to have the capability to diagnose DCM with the most excellent specificity and sensitivity. Meanwhile, we found that *ASNP* and *LUM* were upregulated in DCM compared to normal samples, whereas *IL10* and *CD163* were downregulated. In addition, we also conducted GO and KEGG enrichment analyses of DEGs, and the results showed that DEGs were significantly enriched in immune-related and energy metabolism-related signaling pathways. Recent research was also revealed that immunoadsorption could improve LV function in DCM (18). We further used the CIBERSORT algorithm to explore the landscape of immune cell infiltration between different tissues and correlated the abundance of immune cell infiltration with the expression of hub genes. The results showed that activated NK cells and eosinophils as hub immune cells were identified in DCM myocardial tissues. Moreover, the correlation between immune cells and biomarkers showed that *ASPN* was negatively correlated with activated NK cells, *LUM* was positively correlated with eosinophils, and *CD163* was negatively correlated with activated NK cells. Finally, we explored prediction efficiency of hub genes and hub immune cells, and the combined eosinophils and *CD163* have the perfect capability to diagnose DCM.

With the development of genetics, the concept of genotype-guided therapy is promising. At present, different treatment strategies should be adopted for different genetic changes (19). Combined with relevant conclusions in this study, we are expected to carry out personalized management of precision medicine for each DCM patients. For example, it has been reported that *SCN5A* gene is related to sodium channel. Standard therapy is relatively ineffective for patients with *SCN5A* gene mutation, but sodium channel blocking drugs can significantly improve their hemodynamics (20). In addition, the molecular changes involved in P38 MAP were explored in a research based on *LMNA* gene mutation mice, and p38 inhibitors were used to treat mice, showing an inhibitory effect on LV remodeling (21). It is worth noting that there have been two iPSC-CM experiments *in vitro* to correct the *PLN* and *R14Del* genes, which can completely reverse the cardiac expansion phenotype *in vitro* (22, 23). In four hub genes, *CD163* was associated with M2 macrophages. In an immunohistochemical study of 163 patients with DCM, a significant correlation between *CD163* and collagen area was suggested by multivariate regression analysis, suggesting a correlation between M2 macrophages and collagen formation, and suggesting that *CD163* expression may be related to DCM (23). Interestingly, *ASPN* increased H9C2 cardiomyocyte apoptosis with downregulation of Bcl-2, upregulation of transforming growth factor-β1, Bax, collagen III, fibronectin, and phosphorylation of smad2 and smad3 (24). It indicates that the role of *ASPN* in cardiomyocyte has been preliminarily confirmed, and *ASPN* may be a potential promising biomarker for heart failure (25). Excitingly, interleukin (IL)-10 in cardiovascular disease has been widely studied. Jung et al. (26) indicated that *in vivo* infusion of IL-10 post-MI improves the LV microenvironment to dampen inflammation and facilitate cardiac wound healing (26). In the study of Jiao et al. (27), they used flow cytometry to detect 35 DCM patients and 44 healthy controls, and conclusion showed IL-10-producing Bregs were significantly lower in DCM patients compared with healthy controls (27). Unlike above three hub genes, the role of *LUM* in the cardiovascular system is poorly explored. However, *LUM* has been widely studied in tumors. Some studies have shown that *LUM* is involved in tumor inflammatory signal transduction, which affects the development of tumors by binding to integrin subunits such as β2, α, and αL on polymorphonuclear leukocytes (28). This suggests that *LUM* gene may also be a new direction for future research in DCM. Immune cells in the myocardial microenvironment are the key of immunotherapy. In this study, it was found that activated NK cells and eosinophils may play an important regulatory role in DCM. It has been found that in the pathological process of DCM, activated NK cells and neutrophils play a crucial role. Kanda et al. (29) investigated abnormalities in NK cells in the myocardium and circulating blood of patients with DCM, and 12 normal control subjects (29). They revealed NK cell subsets of DCM patients have abnormal functions, which may be related to the pathogenesis. Although there are few studies on the correlation between eosinophils and DCM, it is interesting to note that a subtype of DCM may be a sequela of hypereosinophilic heart disease (30).

In summary, we explored predictive biomarkers and immune microenvironment in DCM tissues. The study still has some limitations. First, the hub genes must be verified by more *in vitro* and *in vivo* experiments to further explain the potential mechanism of DCM. In addition, although the CIBERSORT algorithm is a classical bulk-RNA deconvolution tool, further experiments are needed to clarify the status of the immune cell infiltration.

## Conclusion

The four hub genes *ASPN, CD163, IL10*, and *LUM* may have potential predictive ability for DCM, and especially *CD163* showed most robust efficacy. Furthermore, activated NK cells and eosinophils may relate to the occurrence of DCM.

## Data Availability Statement

The datasets presented in this study can be found in online repositories. The names of the repository/repositories and accession number(s) can be found in the article/Supplementary Material.

## Ethics Statement

The animal study was reviewed and approved by Institutional Ethics Committee of Affiliated Hospital of Nantong University.

## Author Contributions

QZ, MF, and XC conducted statistical analysis, carried out the experiments, and drafted the article. HP and MP contributed to reviewing the article. MP, YS, and HG edited and revised the article. All authors contributed to manuscript revision, read, and approved the submitted version.

## Funding

This work was supported by grants from the National Natural Science Foundation of China (No. 82000380) and the Municipal Natural Science Foundation of Nantong (No. MS12021071).

## Conflict of Interest

The authors declare that the research was conducted in the absence of any commercial or financial relationships that could be construed as a potential conflict of interest.

## Publisher's Note

All claims expressed in this article are solely those of the authors and do not necessarily represent those of their affiliated organizations, or those of the publisher, the editors and the reviewers. Any product that may be evaluated in this article, or claim that may be made by its manufacturer, is not guaranteed or endorsed by the publisher.

## Supplementary Material

Supplementary Figure 1 ROC analysis of combined hub immune cells with *CD163*. **(A)** ROC analysis of each hub immune cell in screening dataset. **(B)** ROC analysis of combined hub immune cells with *CD163* in screening dataset. ROC, receiver operating characteristic.

Supplementary File 1 A list of DEGs.
